# Genome-wide long noncoding RNA and mRNA expression profiles demonstrate associations between exposure to inorganic elements and the risk of developing hepatocellular carcinoma

**DOI:** 10.1186/s12920-021-00927-w

**Published:** 2021-03-18

**Authors:** Zan-Xi Fang, Jian-Jun Niu, Ping-Guo Liu, Yong Lin

**Affiliations:** 1 Center of Clinical Laboratory, Zhongshan Hospital, School of Medicine, Xiamen University, 209 Hubin South Road, Xiamen, 361004 Fujian Province China; 2 Department of Hepatobiliary Surgery, Zhongshan Hospital, Medical College of Xiamen University, Xiamen, China; 3 Fujian Provincial Key Laboratory of Chronic Liver Disease and Hepatocellular Carcinoma, Xiamen, China; 4 Institute of Infectious Disease, School of Medicine, Xiamen University, Xiamen, China

**Keywords:** LncRNA, Hepatocellular carcinoma, Hepatitis B virus, Microarray analysis

## Abstract

**Background:**

Long noncoding RNAs (lncRNAs) are closely associated with the development of hepatocellular carcinoma (HCC). The present study conducted a genome-wide microarray analysis and qPCR validation to obtain comprehensive insights into this issue.

**Methods:**

Thirty male HCC patients with chronic HBV infection were included in the present study. Primary HCC tissue and normal tissue were collected. Double-stranded complementary DNA synthesized from 10 pairs of samples was labeled and hybridized to a microarray chip. Further analyses, such as hierarchical clustering, gene ontology (GO) and pathway analyses, were performed. In addition, qPCR validation was performed on tissue samples and additional serum samples.

**Results:**

The microarray analysis identified 946 upregulated and 571 downregulated lncRNAs and 1720 upregulated and 1106 downregulated mRNAs. Among these RNAs, ENST00000583827.1 (fold change: 21) and uc010isf.1 (fold change: 18) were the most over- and underexpressed lncRNAs in the HCC tissues, respectively. For the mRNAs, KIF20A (fold change: 26) and HEPACAM (fold change: 50) were the most over- and underexpressed in the HCC tissues, respectively. The GO analysis demonstrated that the most differentially expressed mRNAs were related to the response of metal ions. The pathway analysis also suggested that the most enriched pathway was mineral absorption.

**Conclusions:**

The subsequent qPCR validation exhibited high consistency with the microarray analysis, except for three lncRNAs. The qPCR analysis also demonstrated that TCONS_00008984 had a 767-fold overexpression level in HCC tissues when compared with normal tissues, and this finding was confirmed in the serum samples; therefore, TCONS_00008984 has the potential to serve as a diagnostic marker or prognostic indicator. The GO and pathway analyses indicated that exposure to inorganic elements may be involved in HCC risk.

**Supplementary Information:**

The online version contains supplementary material available at 10.1186/s12920-021-00927-w.

## Background

Primary hepatocellular carcinoma (HCC) has been qualified as the most common liver malignancy worldwide, and its high fatality rate has placed a heavy burden on both patients and the healthcare system. Annually, half a million incidences of HCC emerge, and 1% of total deaths are closely related to HCC worldwide. With the progress of medical science, chronic infection with hepatitis B virus (HBV) and hepatitis C virus (HCV) and the potential development of cirrhosis and fibrosis have been acknowledged as the major risk factors for developing HCC and account for approximately 80% of HCC incidence [1, 2]. Based on previously acquired evidence, it can be concluded that most regions with a high prevalence of HCC show an overlap with a high prevalence of HBV or HCV infection. More precisely, among these hepatitis virus-related HCC patients, HBV is responsible for 75–80% of virus-related HCC cases while HCV accounts for the remaining 10–20% [3]. China has the largest population with chronic HBV infection in the world, and with the efforts made in immunization programs and chronic HBV patient management, the prevalence rate of HBV has dropped from 9.8 to 7.2% in the general population [4]. However, due to the absence of ideal therapy to eradicate chronic HBV infection, a large population still suffers from chronic HBV infection and its possible complications, and this specific population is at high risk of developing HCC. Moreover, approximately half of HCC cases worldwide have been reported in China, which has been recognized as a pressing public health issue [5].

Statistical findings have indicated that males have an approximately four-fold higher risk of developing HCC than their female counterparts. The reason behind these phenomena is that males have a higher chance of being chronically infected with hepatitis viruses based on the disease transmission route as well as factors such as alcohol consumption, smoking, and elevated iron level. Even within the population with chronic infection of viral hepatitis, men still maintain a higher incidence of HCC than women, and reports have also observed higher levels of aflatoxin markers in blood samples collected from male subjects compared with female subjects [6]. Therefore, males with chronic HBV infection in China can definitely be identified as a high-risk population for developing HCC, and etiological studies of HCC targeted specifically on this population are urgently needed to provide scientific evidence for intervention and prevention.

Noncoding sequences represent approximately 97.2% of the human genome [7]. At inception, noncoding regions are considered nonfunctional ballast that fills in the genome between coding genes [8]. However, due to the rapid development of high-throughput sequencing methods and microarray technology, a large number of noncoding RNAs, including long noncoding RNAs (lncRNAs), have been identified in mammalian cells. To date, a wide variety of biological functions of lncRNAs have been discovered, including X chromosome inactivation [9] and gene expression regulation [10], and more importantly, lncRNAs have a direct impact on mRNA stability [11]. A number of investigations have been conducted to study the association between lncRNAs and the risk of developing HCC, and they have primarily identified highly upregulated in liver cancer (HULC) lncRNA. HULC is one of the most excessively expressed lncRNAs in HCC tissue, and it is capable of suppressing several microRNAs (miRNAs), including miR-372, and consequently results in an autoregulatory loop in which HULC promotes its own expression [12]. Moreover, the expression of HULC is associated with hepatitis B virus X protein (HBx) in HCC tissue and inversely correlated with the tumor suppressor gene p18 located near HULC on the same chromosome [13]. Therefore, it can be concluded that the expression of HULC is involved in the proliferation of hepatoma cells and disease progression. Although evidence has been obtained by previous investigators, individual studies have mostly focused on particular lncRNAs and applied microarray analyses, which can cover all known lncRNAs, on a limited sample due to the high cost of employing high-throughput lncRNA chips. More importantly, previous studies were not focused on male HCC patients with chronic HBV infection and apparently failed to investigate the impact of HBV infection on the lncRNA expression profile. To further expand the statistical power of such approaches and gain comprehensive insights into the lncRNA expression profile in male HCC patients with chronic HBV infection, we conducted a microarray analysis of 20 paired HCC tissues and adjacent tissues and performed a qPCR validation among 60 paired tissues and 105 blood samples from HBV-related HCC cases and an equal number of chronic HBV infection participants to confirm the results generated from the microarray analysis.

## Materials and methods

### Study participant and sample preparation

During November 2015 to December 2020, we managed to recruit 30 male HCC patients with chronic HBV infection. Patients were included in the present study if they met the following criteria (1) primary HCC confirmed by a pathological report; (2) male aged from 20 to 79 years; (3) before the onset of HCC, patients were diagnosed with chronic HBV infection by ELISA or any diagnostic tests; (4) they are residents who lived in Xiamen for over 10 years. Patients were ineligible if they met any of these conditions: (1) liver disorders due to factors other than HBV infection, including parasitosis, fatty liver, excessive alcohol consumption and diabetes; (2) have been diagnosed with any other kind of cancer; (3) hepatitis caused by autoimmune diseases or exposure to chemical toxins; or (4) decline to be enrolled. Primary HCC tissues were obtained from all enrolled study participants during an operation to remove the tumor. Normal tissues which were 5 cm from the tumor edge were also collected simultaneously to serve as the controls. Once these two kinds of liver tissues were removed from the patients, they were transferred to an liquid nitrogen prefreezing RNase-free vials for 5 min, and before the subsequent experiments, tissues were stored at − 78 °C. RNA extraction of tissue samples were conducted by using TRIzol reagent (Invitrogen, MA, USA), which were consistent with the recommended protocol [14]. The absorbance at 260 nm and 280 nm was measured in a spectrophotometer and the 260/280 ratio was calculated to assess the purity and concentration of RNA extracts, as Wilfinger et al. recommended [15]. In order to evaluate the integrity, we also employed standard denaturing agarose gel electrophoresis to assess each RNA extract, and those which have a total volume greater than 8 μg were included in all subsequent analyses.

In addition, 105 male HBV-related HCC patients and the same number of patients with chronic HBV infection were enrolled. Each study participant provided a blood sample of 5 mL for subsequent qPCR validation. The inclusion and exclusion criteria of the male HBV-related HCC patients were the same as those mentioned above. For patients with chronic HBV infection, they were included if following conditions were met: (1) diagnosed with chronic HBV infection by ELISA or any diagnostic tests; (2) male; (3) age matched with HBV-related HCC patients; and (4) residents who lived in Xiamen for over 10 years. The exclusion criteria include: (1) liver disease other than chronic HBV infection; (2) diagnosed with any kind of cancer; and (3) decline to be enrolled in the present study. Blood samples were centrifuged at a speed of 10,000 rpm for 10 min at 4 °C. After centrifugation, the serum supernatant was transferred into an RNase-free tube and stored at − 80 °C for RNA extraction and qPCR analysis.

After a formal hearing, this study was approved by the ethical Committee of Zhongshan Hospital, Xiamen University after a formal hearing and a voting process. The study conformed with the Declaration of Helsinki. During the recruitment, a consent form was given to each study participant before his operation. Investigators have extensively elaborated the purpose, the sample collection procedure, potential risks, privacy protection and the right to withdraw to each participant. Each participants physically singed the consent before enrollment.

### Microarray analysis

RNA extracts from HCC tissues and normal tissues were used to synthesize double stranded complementary DNA (cDNA). After the synthesis, cDNA products were labeled and hybridized to lncRNA + mRNA Human Gene Expression Microarray V4.0 (CapitalBio Corp, Beijing, China) which contains 40,916 human lncRNA probes, 34,235 mRNA probes, and 4974 Agilent control probes [16]. The expression level of each RNA was measured by the corresponding probe for two times.

### Microarray imaging and data analysis

After the completion of microarray analysis, data were subjected to summarization, normalization and quality control by using GeneSpring software version 12.0 (Agilent, CA, USA). Differentially expressed genes were defined as those with a fold change equal or greater than two and a Benjamini–Hochberg corrected *P* value less than 0.05. The expression level of each gene was log 2 transformed and median centered by employing the Multiexperiment Viewer software (Dana-Farber Cancer Institute, MA, USA).

### Gene ontology (GO) and pathway analysis

The differentially expressed mRNAs identified in the comparison analysis were further subjected to GO analysis and pathway analysis based on Kyoto Encyclopedia of Genes and Genomes (KEGG) database were conducted to investigate the enriched GO terms and biological pathways. The detailed procedure of these analyses has been previously reported [17].

### qPCR validation

In total, 18 significantly upregulated and 4 downregulated lncRNAs have been included in the qPCR to validate their expression level in a larger sample size. To be more specific, the expression level of these selected lncRNAs was measured in the samples subjected to microarray analysis and additional paired tissue samples from other 20 subjects. Additionally, 210 paired serum samples were introduced for qPCR validation. RNA extraction and quality assessment of tissue samples were described above. The total RNA of the serum samples was isolated using an RNA Isolation Kit (Axygen Scientific, CA, USA). Reverse transcription was performed using a FastQuant RT Kit with gDNase (Tiangen, Beijing, China). The total reaction volume for PCR was 10 μL that consists of 5 μL of Power SYBR Green PCR Master Mix (Applied Biosystems, CA, USA), 0.25 μL of forward primer and reverse primer each, 0.5 μL of template, and 4 μL of nuclease-free water. PCR was conducted as follows: enzyme activation at 95 °C for 10 min, denaturation at 95 °C for 15 s for 40 cycles, annealing and extension at 60 °C for 1 min, and melting curve analysis was conducted between 60 and 95 °C. Hydroxymethylbilane synthase (HMBS) was employed as the reference gene for qPCR analysis of the tissue samples, and β-actin was used as the reference gene for the serum samples. The primers were as follows: HMBS forward, 5′-CACGATCCCGAGACTCTGCT-3′, and reverse, 5′-TACTGGCACACTGCAGCCTC-3′; β-actin forward, 5′-GGCACCCAGCACAATGAAG-3′, and reverse, 5′-CCGATCCACACGGAGTACTTG-3′. The relative expression level of each lncRNA was calculated by using 2^−∆∆CT^ method.

## Results

### Differential expression of lncRNAs and mRNAs in HCC and normal tissues

**Fig. 1:**
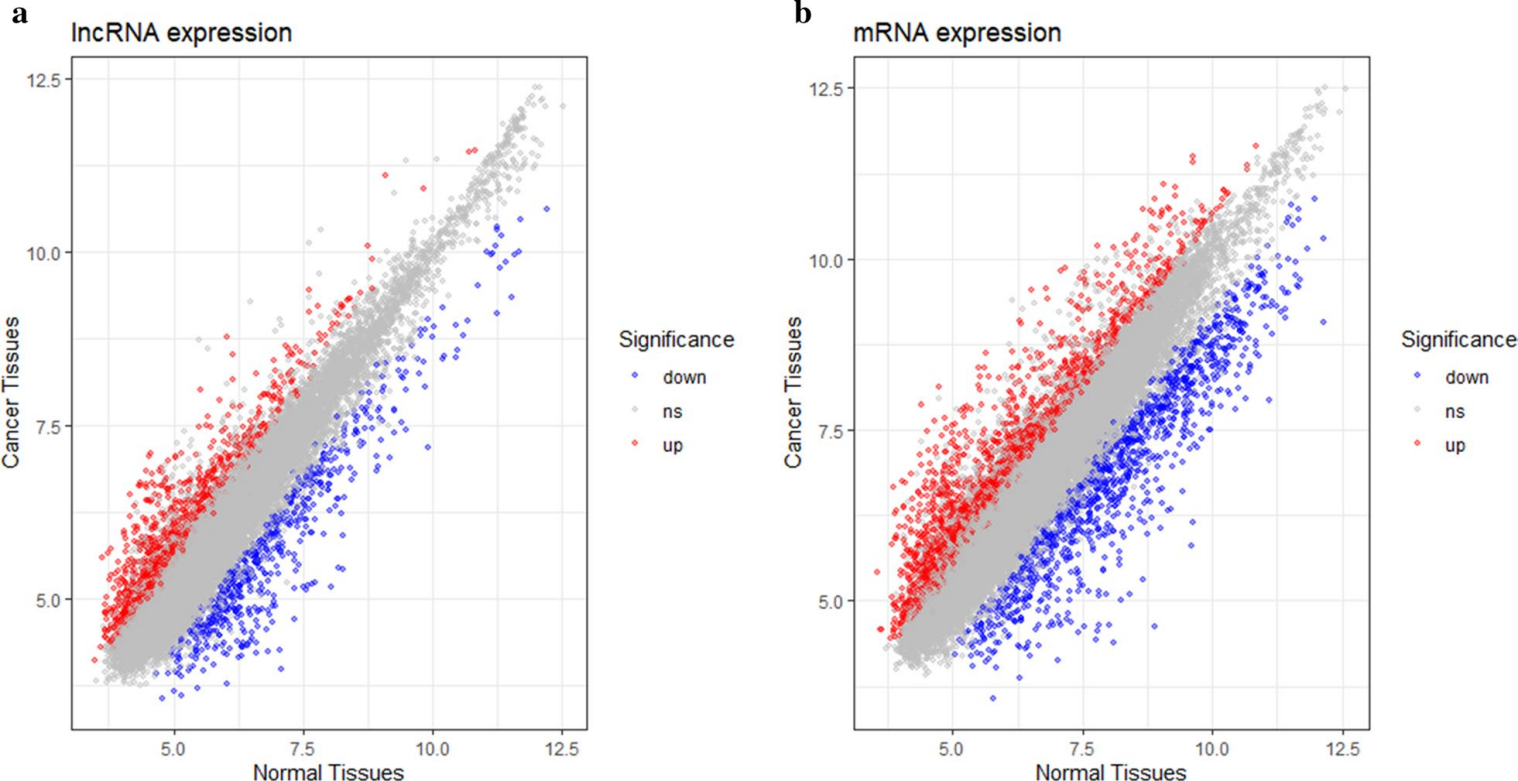
The scatter plot of **a** lncRNA and **b** mRNA expression signals in HCC and normal tissues

**Fig. 2:**
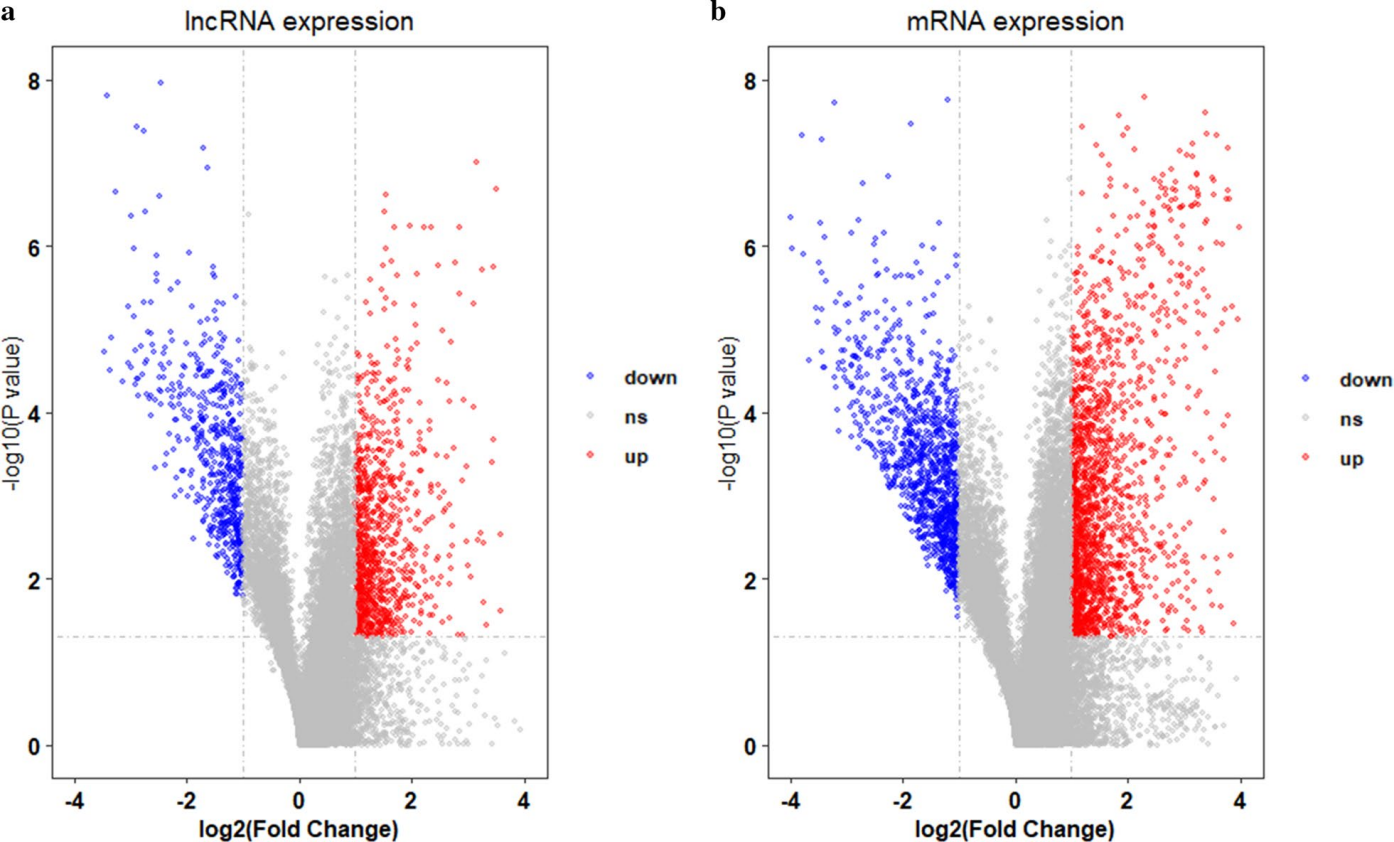
The volcano plot of **a** lncRNA and **b** mRNA expression signals in HCC and normal tissues

As stated in the Materials and Methods section, the microarray we employed in the present study covers 40,916 human lncRNA probes. Among all tested lncRNAs, lncRNAs with more than twofold expression changes (*P* < 0.05) and at least three biological replicates were considered differentially expressed. Based on these criteria, the microarray analysis identified 946 upregulated and 571 downregulated lncRNAs. The top 30 differentially expressed lncRNAs are listed in Table 1. ENST00000583827.1 (fold change: 21) and uc010isf.1 (fold change: 18) were the most over- and underexpressed lncRNAs in the HCC tissues, respectively. The expression level was detected for 34,235 mRNAs, and we found 1720 upregulated and 1106 downregulated mRNAs in accordance with the same criteria applied for screening lncRNAs. KIF20A (fold change: 26) and HEPACAM (fold change: 50) were the most over- and underexpressed mRNAs in HCC tissues compared with their normal counterparts, respectively. The scatter plot of differentially expressed lncRNAs and mRNAs is shown in Fig. 1, and the volcano plot is shown in Fig. 2.

**Table 1:** The top 30 differentially expressed lncRNAs and mRNAs between HCC and normal tissues

Rank	lncRNA			mRNA		
	GeneSymbol	Regulation	Fold change	GeneSymbol	Regulation	Fold change
1	ENST00000583827.1	Up	21.11	HEPACAM	Down	49.56
2	uc010isf.1	Down	17.76	CLEC4G	Down	44.74
3	XR_109840.2	Down	16.41	CXCL14	Down	38.37
4	TCONS_00023687	Down	15.08	COLEC10	Down	35.43
5	ENST00000596540.1	Up	12.01	SLC25A47	Down	31.15
6	RNA147023|p0127_imsncRNA205	Up	11.88	KIF20A	Up	26.44
7	ENST00000521666.1	Up	11.30	HHIP	Down	26.17
8	ENST00000559280.1	Up	10.96	CLEC1B	Down	23.87
9	NR_027462.1	Down	10.94	CYP2E1	Down	23.85
10	ENST00000591629.1	Up	10.92	TOP2A	Up	22.89
11	ENST00000437781.1	Up	10.75	PLIN2	Down	22.72
12	ENST00000568523.1	Down	10.56	THBS4	Up	22.61
13	TCONS_00005551	Down	10.26	MEP1A	Up	22.53
14	ENST00000597260.1	Down	10.05	CYP26A1	Down	22.18
15	TCONS_00012039	Up	9.96	SPP1	Up	19.81
16	ENST00000597655.1	Up	9.74	AKR1B10	Up	19.44
17	TCONS_00008984	Up	9.73	HPD	Down	18.85
18	ENST00000451264.1	Down	9.60	HEPN1	Down	18.42
19	ENST00000560278.1	Up	9.47	MELK	Up	18.35
20	ENST00000608652.1	Up	9.39	BUB1	Up	17.82
21	ENST00000600679.1	Up	8.98	FCN2	Down	17.76
22	ENST00000609879.1	Up	8.94	NEB	Up	17.22
23	XR_158989.2	Down	8.73	MKI67	Up	17.19
24	ENST00000584351.1	Up	8.62	CD5L	Down	17.07
25	ENST00000432120.1	Up	8.58	SPINK1	Up	17.01
26	ENST00000415810.1	Up	8.35	DEPDC1	Up	16.93
27	TCONS_00023870	Down	8.19	HHIP	Down	16.84
28	TCONS_00023860	Down	8.19	BMP10	Down	16.45
29	TCONS_00016038	Up	8.00	MAGEA2B	Up	16.43
30	ENST00000566797.1	Down	7.91	CDKN3	Up	15.93

**Fig. 3:**
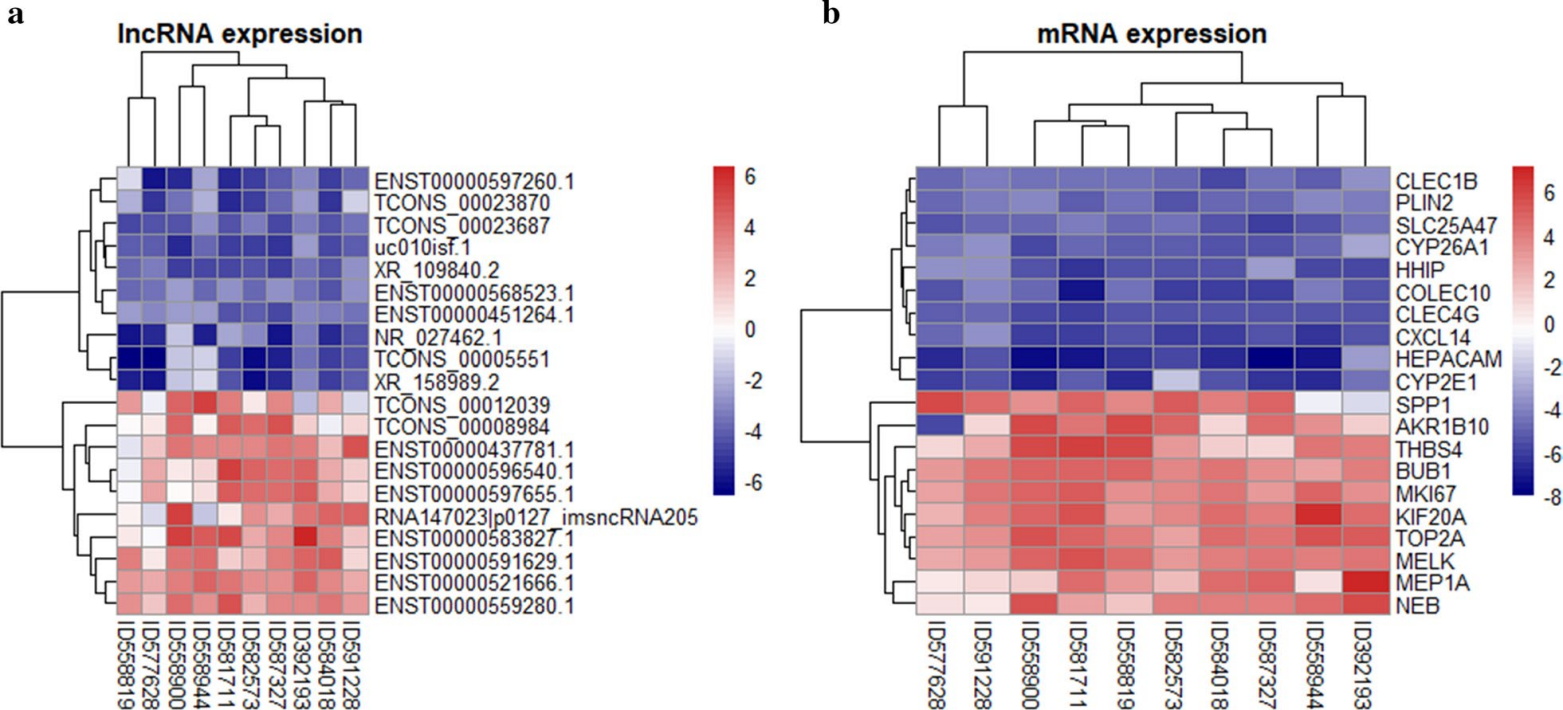
Top 20 differentially expressed **a** lncRNA and **b** mRNA in HCC and normal tissues

A hierarchical clustering analysis was conducted to group lncRNAs and mRNAs based on the expression level of all RNAs determined in the microarray analysis, allowing us to hypothesize the relationship among samples. Figure 3 shows the top 20 differentially expressed lncRNAs (Fig. 3a) and mRNAs (Fig. 3b) in the microarray experiment.

### Gene ontology (GO) and pathway analysis results

**Fig. 4:**
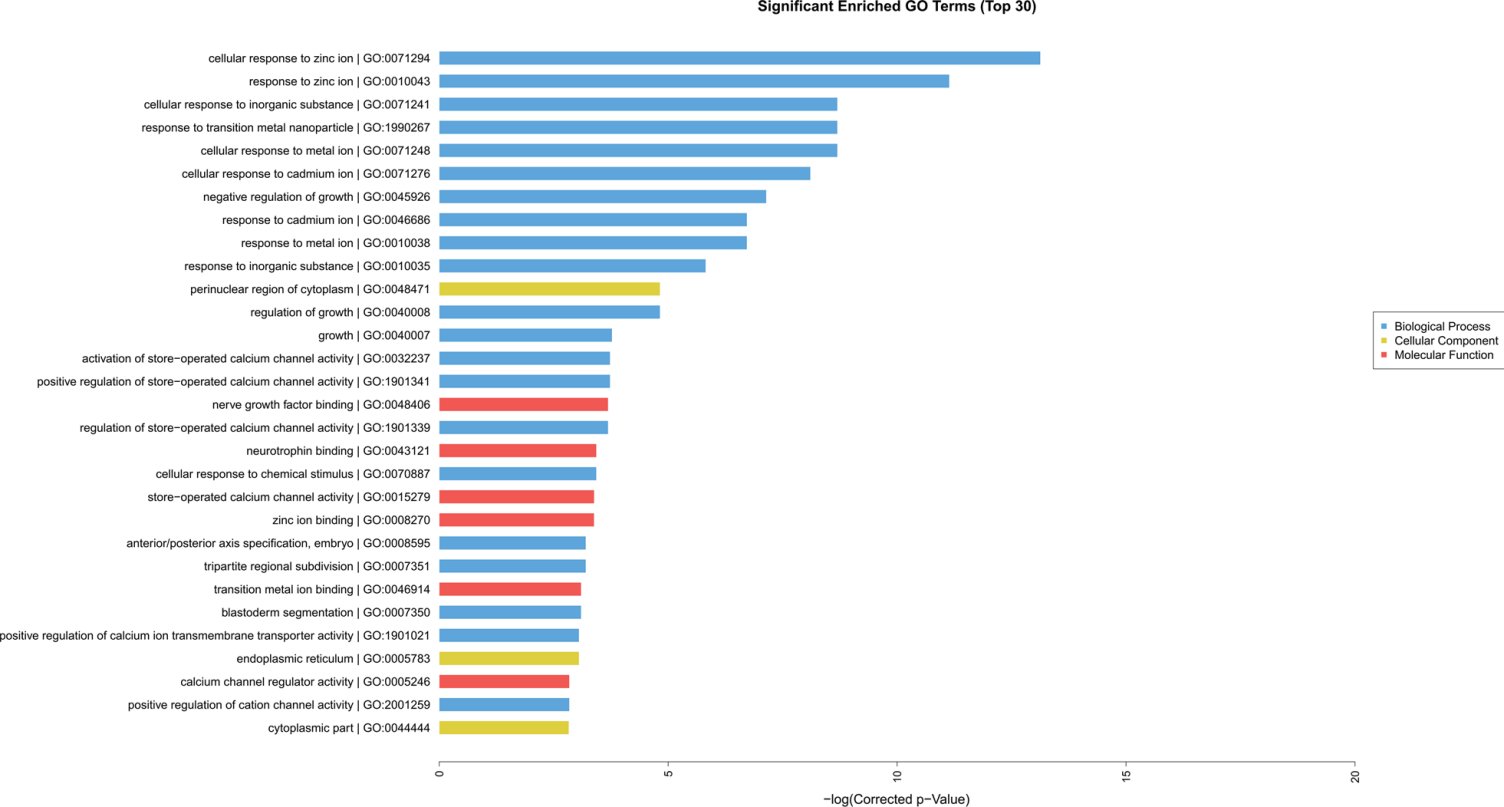
GO enrichment analysis of differentially expressed mRNAs

A GO analysis was conducted to gain insights into the potential functions of the differentially expressed host genes in HCC tissues when compared with normal counterparts. Differentially expressed mRNAs from the microarray analysis were classified into different functional categories, including biological process, cellular component, and molecular function. Figure 4 lists the top 30 enriched GO terms, and most of the enriched GO terms were classified as biological process. More importantly, the top 10 enriched GO terms were all involved in biological processes, with most of the top 10 enriched GO terms related to the response to metal ions. Only three cellular components ranked in the top 30, and the most enriched term in this category was the perinuclear region of cytoplasm (GO: 004008). For molecular function, six of the components were listed in the top 30 enriched GO terms, with the most enriched GO term nerve growth factor binding (GO: 0048406).

**Fig. 5:**
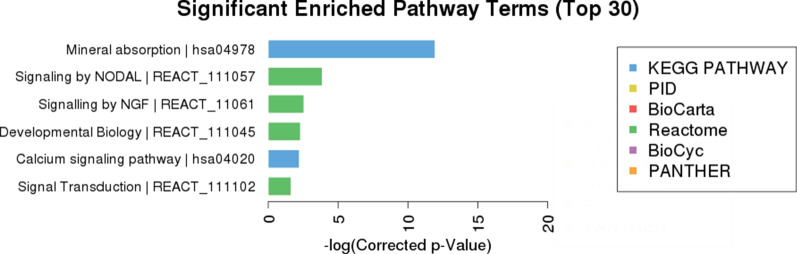
Pathway analysis of differentially expressed mRNAs

Pathway analysis was used to investigate the involved biological pathways of the differentially expressed mRNAs in HCC carcinogenesis. The analysis suggested that the differentially expressed mRNAs in HCC tissue mainly participated in mineral absorption (hsa04978), signaling by NIDAL (REACT_111057), signaling by NGF (REACT_11061), development biology (REACT_111045), calcium signaling pathway (hsa04020) and signal transduction (REACT_111102). The detailed results of the pathway analysis are shown in Fig. 5.

### LncRNA-mRNA correlation and target prediction

A correlation analysis between the differentially expressed lncRNAs and mRNAs was conducted, and for each pair of genes, Pearson’s correlation was calculated. The significantly correlated pairs that had a Pearson correlation coefficient greater than 0.99 and a *P* value less than 0.05 are listed in Additional file 1. Target prediction was conducted using blat tools to compare the full sequence of the lncRNA with the 3′ UTR of its coexpressed mRNAs with the default parameter settings, and the results can be found in Additional file 2.

### qPCR validation

**Fig. 6:**
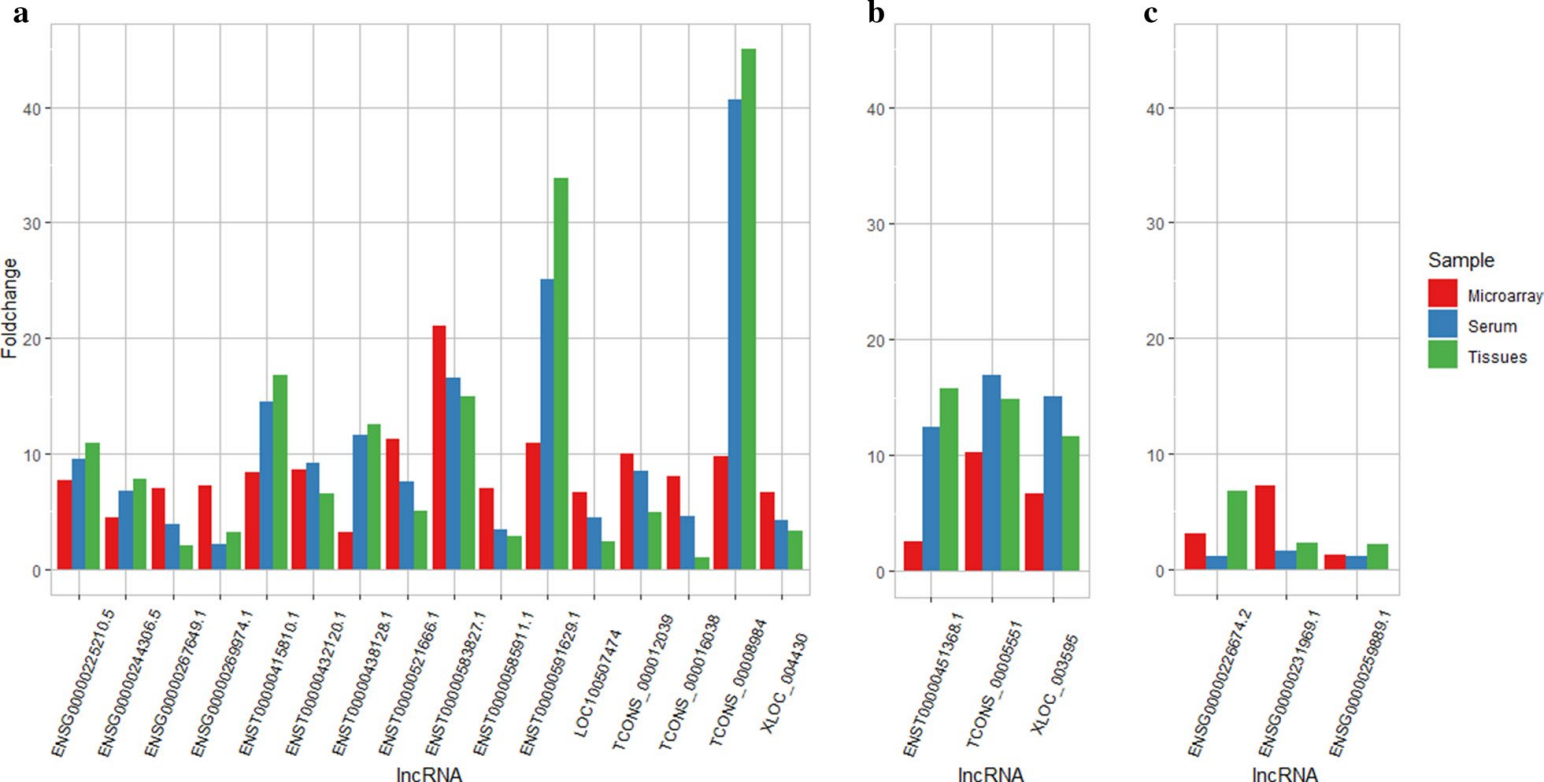
Fold change of 22 selected lncRNAs in **a** up-regulated, **b** down-regulated and **c** non-significant

The expression levels of 18 significantly upregulated and 4 downregulated lncRNAs, which were selected based on the fold change and biological replicates, were determined by qPCR of 30 pairs of tissues collected from HCC patients and blood samples from HBV-related HCC patients and patients with chronic HBV infection. The criteria were the same as in the microarray analysis. The qPCR results of the tissue and serum samples are presented in Table 2 along with the results generated from microarray analysis of the corresponding lncRNAs. Three discrepancies were observed between the qPCR and microarray analysis. According to the microarray analysis, the expression level of ENSG00000259889.1 was significantly elevated in HCC tissue compared with normal tissue; however, the qPCR results of the tissue and serum samples demonstrated that the difference was not significant. Nonsignificant results were also observed in ENSG00000231969.1 and ENSG00000226674.2 when analyzing the serum samples. The remaining qPCR results of 19 lncRNAs all showed good alignment when compared with the microarray analysis, suggesting the reliability of the microarray analysis in the present study. What was striking about the qPCR results was that TCONS_00008984 had a 769-fold expression level in HCC tissue compared with normal tissue. Although the fold change was reduced to 41 in the serum samples with a larger sample size, it still has the potential to be a diagnostic biomarker for the early detection of HCC. Moreover, the expression level of ENST00000591629.1 was elevated 34 times in HCC tissue and 25 times in the serum samples according to qPCR results. A detailed comparison of the microarray analysis and qPCR validation is shown in Table 2 and Fig. 6. These highly altered lncRNAs could possibly be involved in the carcinogenesis of HCC.

**Table 2:** The comparison on qPCR validation results of 22 lncRNAs and microarray data

lncRNA	Fold change			Regulation		
Sample	Microarray	Tissue	Serum	Microarray	Tissue	Serum
ENST00000585911.1	7.04	2.82	3.45	Up	Up	Up
ENST00000591629.1	10.92	33.81	25.12	Up	Up	Up
ENST00000415810.1	8.35	16.79	14.45	Up	Up	Up
TCONS_00005551	10.26	14.83	16.90	Down	Down	Down
ENST00000438128.1	3.14	12.55	11.59	Up	Up	Up
ENST00000521666.1	11.30	5.07	7.58	Up	Up	Up
TCONS_000016038	8.00	1.02	4.55	Up	Up	Up
ENST00000432120.1	8.58	6.49	9.14	Up	Up	Up
ENST00000451368.1	2.54	15.78	12.50	Down	Down	Down
TCONS_000012039	9.96	4.91	8.50	Up	Up	Up
ENST00000583827.1	21.11	14.96	16.55	Up	Up	Up
TCONS_00008984	9.73	768.94	40.68	Up	Up	Up
ENSG00000269974.1	7.25	3.13	2.11	Up	Up	Up
ENSG00000231969.1^a^	7.20	2.21	1.56	Down	Down	Non-significant
ENSG00000267649.1	7.04	2.07	3.87	Up	Up	Up
LOC100507474	6.70	2.37	4.50	Up	Up	Up
XLOC_004430	6.65	3.27	4.21	Up	Up	Up
ENSG00000226674.2^a^	3.09	6.75	1.14	Up	Up	Non-significant
XLOC_003595	6.66	11.60	15.12	Down	Down	Down
ENSG00000225210.5	7.65	10.94	9.51	Up	Up	Up
ENSG00000259889.1^a^	1.23	2.19	1.13	Up	Non-significant	Non-significant
ENSG00000244306.5	4.40	7.79	6.72	Up	Up	Up

^a^Contradicted results between microarray analysis, tissue, and serum samples

## Discussion

In recent years, the dysregulation of lncRNA expression profiles has been studied widely to elucidate the underlying molecular mechanisms that contribute to the development of human cancers [18]. With the rapid development of microarray technology and a sustained increase in the coverage of human lncRNAs, a certain number of lncRNAs have been found to be associated with carcinogenesis. Microarray technology possesses several advantages, including high-throughput properties covering the whole human genome, high sensitivity, and ability to perform lncRNA-mRNA correlation analyses [19]. However, due to the high cost of employing microarray chips, studies are often conducted using a limited sample size. Under such conditions, the credibility of lncRNAs may be affected by random errors or outliers. Therefore, it is imperative to conduct qPCR validation on a larger sample size to verify the results generated by microarray analysis. In the present study, we first investigated the lncRNA and mRNA expression profiles in 10 HCC tissues and normal tissues collected from 10 male HCC patients with chronic HBV infection. Compared with normal tissue, we identified 946 upregulated and 571 downregulated lncRNAs. Moreover, 1720 upregulated and 1106 downregulated mRNAs were found by the same criteria. A qPCR assay was performed for 30 pairs of tissues along with 210 paired serum samples to validate the expression level of 22 selected lncRNAs. Overall, the results between different methods showed high consistency except for one lncRNA, suggesting the reliable performance of microarray analysis.

We conducted a GO enrichment analysis to investigate the potential functions and pathways of differentially expressed mRNAs. Surprisingly, the enriched GO terms were mostly involved in the metal ion response, including zinc and cadmium. The results supported evidence from the study conducted by Ebara et al. [20] which observed an accumulation of copper-metallothionein (Cu-MT) in HCC tissue and significantly higher zinc metallothionein (Zn-MT) levels in surrounding normal liver tissue. Zn has potent antioxidative abilities, and Zn deficiency leads to enhanced oxidative stress and increased production of inflammatory cytokines. Oxidative stress may trigger a harmful inflammatory response and is responsible for cancer [21]. The subjects we included in the present study had undergone chronic HBV infection before the onset of HCC, and HBV was capable of integrating into the host genome during the course of infection, consequently leading to oncogene activation, tumor suppressor gene inactivation, or other genome instability [22]. Interestingly, HBV integration can be found in both HCC tissue and normal tissue [23]. However, next-generation sequencing revealed that viral integration occurred more frequently in HCC tissue than in paired nontumor tissues [24]. We assume that the elevated integration level and chronic inflammation are able to disrupt the zinc/copper ratio in liver tissue and consequently produce free radical formation and further induce hepatocyte damage. Unlike Cu and Zn, cadmium (Cd) has been considered a hazardous element to humans and is classified as a human carcinogen [25]. This toxic element is widely distributed in human organs after absorption, with the major portion of the body burden located in the liver and kidney. By employing a rat liver epithelial cell line, a previous study observed Cd-induced malignant transformation and significantly downregulated the expression of apolipoprotein E (ApoE), which was recently established as a suppressor of cell invasion [26]. The source of exposure to Cd can vary; one of the most common sources is smoking due to the high concentrations of Cd in tobacco plants, and despite efforts towards tobacco control, half of Chinese males were smokers according to a recent survey. Moreover, due to the absence of environmental regulation in the early stage of Chinese industrial growth, the soil in many areas was contaminated with wastewater, and rice, which is the staple food of the Chinese population, would accumulate high concentrations of Cd. The pathway analysis also supported our hypothesis because the most enriched pathway in differentially expressed mRNA was mineral absorption (hsa04978). Based on the evidence mentioned above, we can conclude that the intake of some specific inorganic elements was involved in the development of HCC in the Chinese male population. However, further investigations, such as epidemiological studies focusing on inorganic element exposure, should be conducted to verify our assumption.

KIF20A, a member of the kinesin family, plays a critical role in cytokinesis [27], and more importantly, it has been associated with the development and progression of various kinds of human cancers. In vitro experiments conducted by Gasnereau et al. [28] showed that the product of KIF20A mRNA MKlp2 was highly elevated in human hepatoma cell lines but could not be detected in normal human hepatocytes. The essential functions of KIF20A are associated with cell adhesion, spreading, migration and proliferation. Other individual studies employing RNA-silencing methods yielded conclusive results, and the cell viability and invasion capacity were significantly suppressed in cancer cell lines [29, 30]. The findings of previous studies broadly supported the microarray analysis results of the present study, which demonstrated 26-fold overexpression of KIF20A in HCC tissues compared with normal tissues. On the other hand, the key molecule for the inhibition of migration and cell growth, HEPACAM, was found to be 50-fold underexpressed in HCC tissues. Treatment with HEPACAM-overexpressing adenovirus in a cancer cell line was able to promote inhibitory effects on cell proliferation, viability and protein expression [31]. Based on the evidence obtained from an in vitro experiment, the mechanism underlying the inhibitory effects caused by HEPACAM was the inhibition of the Wnt/β-catenin signaling pathway, which is considered a cancer-related pathway if aberrantly activated [32]. The results reflected high activation of cell migration and proliferation in malignant liver hepatocytes.

In the microarray analysis, we identified a large number of dysregulated lncRNAs that have not yet been reported in similar studies. With the subsequent qPCR validation conducted on the microarray analysis samples, twice the number of independent samples and 210 serum samples, we observed high consistency between the three experiments. Among the differentially expressed lncRNAs, TCONS_00008984 exhibited astonishing elevation in HCC tissue, with 769-fold overexpression. Notably, this extremely high expression was not caused by a few outliers but rather can be attributed to a consistent increase in qPCR validation. The fold change of this particular lncRNA remained high, with 41-fold overexpression in the serum samples of HCC cases when compared with patients with chronic HBV infection. Given the extremely high elevation and absence of outliers, TCONS_00008984 has the potential to serve as a diagnostic marker or prognostic indicator, although its biological function remains unclear. An epidemiological study focusing on the association between HCC clinical features and the expression level of TCONS_00008984 should be conducted to assess its ability to serve as a biomarker. In vitro experiments are essential to revealing its underlying mechanism in the carcinogenesis of HCC and should also be performed.

## Conclusions

The present study detected many differentially expressed lncRNAs and mRNAs by comparing HCC tissues and normal tissues via a genome-wide microarray analysis, and the subsequent qPCR validation showed high consistency. GO enrichment and pathway analyses revealed that the mRNAs related to the inorganic ion response were involved in carcinogenesis, especially those associated with Zn and Cd. These findings suggested that exposure to some specific inorganic elements may possibly be involved in the risk of developing HCC; thus, such exposure should not be neglected. Epidemiological studies should be implemented to investigate the synergistic effect between inorganic element exposure and chronic HBV infection, and subsequent interventions should be enhanced to reduce the incidence of HCC.

## Supplementary Information

**Additional file 1.** LncRNA-mRNA correlation analysis.

**Additional file 2.** LncRNA target prediction.

## Data Availability

The datasets used and/or analyzed during the current study have been deposited in NCBI’s gene expression omnibus (GEO) database with an accession number of GSE166705 (https://www.ncbi.nlm.nih.gov/geo/query/acc.cgi?acc=GSE166705).

## Abbreviations

HCC: Hepatocellular carcinoma
HBV: Hepatitis B virus
HCV: Hepatitis C virus
lncRNA: Long non-coding RNA
HULC: Highly-up-regulated in liver cancer
HBx: Hepatitis B virus X protein
cDNA: Complementary DNA
GO: Gene Ontology
KEGG: Kyoto Encyclopaedia of Genes and Genomes
HMBS: Hydroxymethylbilane synthase
Zn: Zinc
Cd: Cadmium
ApoE: Apolipoprotein E
